# Evaluating ChatGPT Responses on Atrial Fibrillation for Patient Education

**DOI:** 10.7759/cureus.61680

**Published:** 2024-06-04

**Authors:** Thomas J Lee, Daniel J Campbell, Abhinav K Rao, Afif Hossain, Omar Elkattawy, Navid Radfar, Paul Lee, Julius M Gardin

**Affiliations:** 1 Department of Medicine, Rutgers University New Jersey Medical School, Newark, USA; 2 Otolaryngology–Head and Neck Surgery, Thomas Jefferson University Hospital, Philadelphia, USA; 3 Department of Medicine, Trident Medical Center, Charleston, USA; 4 Department of Medicine/Division of Cardiology, Rutgers University New Jersey Medical School, Newark, USA

**Keywords:** atrial fibrillation, healthcare technology, patient education, artificial intelligence, chatgpt

## Abstract

Background

ChatGPT is a language model that has gained widespread popularity for its fine-tuned conversational abilities. However, a known drawback to the artificial intelligence (AI) chatbot is its tendency to confidently present users with inaccurate information. We evaluated the quality of ChatGPT responses to questions pertaining to atrial fibrillation for patient education. Our analysis included the accuracy and estimated grade level of answers and whether references were provided for the answers.

Methodology

ChatGPT was prompted four times and 16 frequently asked questions on atrial fibrillation from the American Heart Association were asked. Prompts included Form 1 (no prompt), Form 2 (patient-friendly prompt), Form 3 (physician-level prompt), and Form 4 (prompting for statistics/references). Responses were scored as incorrect, partially correct, or correct with references (perfect). Flesch-Kincaid grade-level unique words and response lengths were recorded for answers. Proportions of the responses at differing scores were compared using the chi-square analysis. The relationship between form and grade level was assessed using the analysis of variance.

Results

Across all forms, scoring frequencies were one (1.6%) incorrect, five (7.8%) partially correct, 55 (85.9%) correct, and three (4.7%) perfect. Proportions of responses that were at least correct did not differ by form (p = 0.350), but perfect responses did (p = 0.001). Form 2 answers had a lower mean grade level (12.80 ± 3.38) than Forms 1 (14.23 ± 2.34), 3 (16.73 ± 2.65), and 4 (14.85 ± 2.76) (p < 0.05). Across all forms, references were provided in only three (4.7%) answers. Notably, when additionally prompted for sources or references, ChatGPT still only provided sources on three responses out of 16 (18.8%).

Conclusions

ChatGPT holds significant potential for enhancing patient education through accurate, adaptive responses. Its ability to alter response complexity based on user input, combined with high accuracy rates, supports its use as an informational resource in healthcare settings. Future advancements and continuous monitoring of AI capabilities will be crucial in maximizing the benefits while mitigating the risks associated with AI-driven patient education.

## Introduction

Atrial fibrillation (AF) is the most prevalent type of serious heart arrhythmia, affecting millions worldwide and associated with significant morbidity and mortality [1]. Educating patients is crucial for managing AF, ranging from understanding the condition to adhering to treatment plans. According to the National Cancer Institute’s (NCI) Health Information National Trends Survey (HINTS), 84.6% of the U.S. adult population sought health or medical information online in 2022, and this figure is expected to rise [2].

ChatGPT, an artificial intelligence (AI) chatbot from OpenAI, was launched in November 2022 and rapidly gained widespread attention. It evolved from a novel technology into a commonly used tool for internet searches. GPT in ChatGPT stands for Generative Pre-trained Transformer, a family of large language models that use deep learning to generate human-like conversational text. After its launch, ChatGPT reached 1 million users in just five days and exceeded 100 million users within two months [3]. Given its growing popularity and potential for disseminating health information, it is crucial to evaluate the quality and accuracy of ChatGPT’s responses on AF. This study aims to critically assess ChatGPT’s answers to patient questions about AF, focusing on the accuracy, clarity, and suitability of these responses for patient education. Our findings aim to guide cardiologists and healthcare professionals in understanding the potential applications and limitations of AI tools such as ChatGPT in patient education. This understanding is critical for integrating AI tools effectively into patient communication strategies and enhancing overall healthcare delivery.

## Materials and methods

ChatGPT was prompted four times and then asked 16 identical questions derived from the American Heart Association’s (AHA) frequently asked questions on AF [4]. ChatGPT version 3.5 was used for all responses. The specific prompts are detailed in Table 1. The responses from ChatGPT were reviewed and scored as incorrect, partially correct, correct, or perfect (correct with references). Responses were marked as incorrect if they included less than 50% of the information from the AHA responses, or if any of the information was incorrect. Responses were marked as partially correct if the answers included 50% to 99% of the information from AHA and had no incorrect information. Responses were marked as correct if the information from ChatGPT included all information from the AHA answers with any extra information being correct. Responses were marked perfect if the answers met the criteria for correct answers and included the requested references and/or statistics. The proportions of responses at each score level were compared using the chi-square analysis. Tests were performed with a significance level set at p < 0.05.

**Table 1:** Prompts used for each form. Each prompt was entered once before the first question.

Form number	Form name	ChatGPT prompt provided
1	No prompting	No prompting
2	Patient-friendly prompting	I am a patient attempting to learn more about atrial fibrillation. I am going to ask you 16 questions pertaining to atrial fibrillation. Please use the language that would be appropriate for my understanding, but do not compromise on the accuracy of your responses. Be as specific as possible in your answers
3	Physician-level prompting	I am a board-certified physician attempting to learn the most up-to-date information on atrial fibrillation. I am going to ask you 16 questions pertaining to atrial fibrillation. Please use the language that would be appropriate for my expert-level understanding of medical concepts. Be as specific as possible in your answers
4	Prompting for statistics and references	I am going to ask you 16 questions pertaining to atrial fibrillation. For each answer you provide, make sure that you include statistics, numbers, or calculations that are relevant. Your answers should come from published medical literature, which you should cite within your answers

The Flesch-Kincaid (FK) grade level was calculated for each ChatGPT answer [5]. This equation estimates the U.S. grade level that would be required to comprehend an answer and is defined as:

$0.39 (\frac{words}{sentences}) + 11.8 (\frac{syllables}{words})$

Values can range from 0 to 20, which correspond to the reading grade level (e.g., 12 would equal grade level 12). Differences between the forms in grade level and response length were analyzed using a one-way analysis of variance (ANOVA), with p < 0.05 considered statistically significant. Statistical analyses were performed using Prism 10.0.2.

## Results

**Figure 1:**
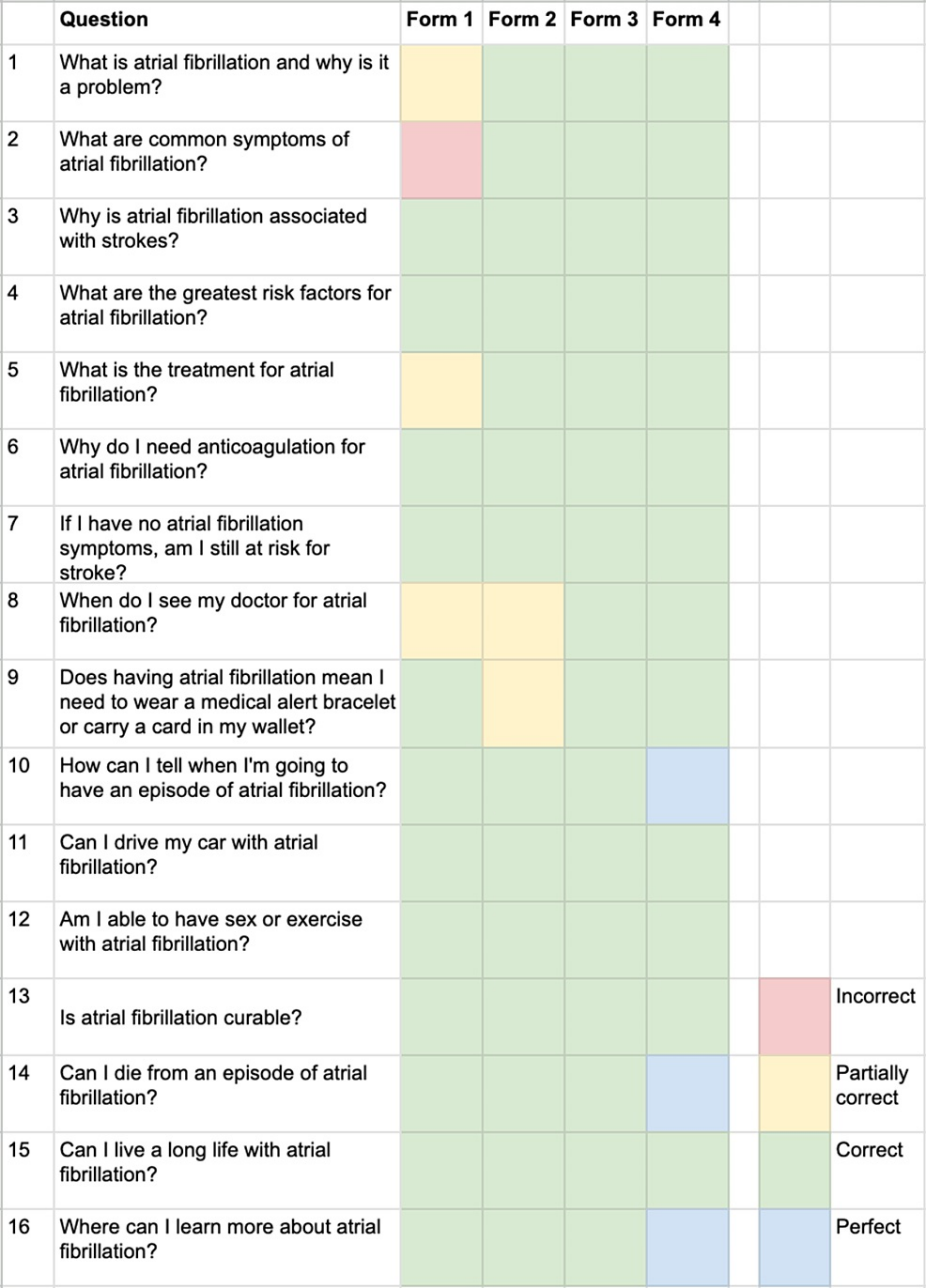
ChatGPT accuracy of answers by form number.

Across all forms, scoring frequencies were one (1.6%) incorrect, five (7.8%) partially correct, 55 (85.9%) correct, and three (4.7%) perfect (Figure 1). Our analysis revealed that the proportions of responses categorized as at least correct (i.e., correct or perfect) did not significantly differ by the form of prompt (p = 0.350). However, Form 4 had significantly more perfect responses (p < 0.023).

**Figure 2:**
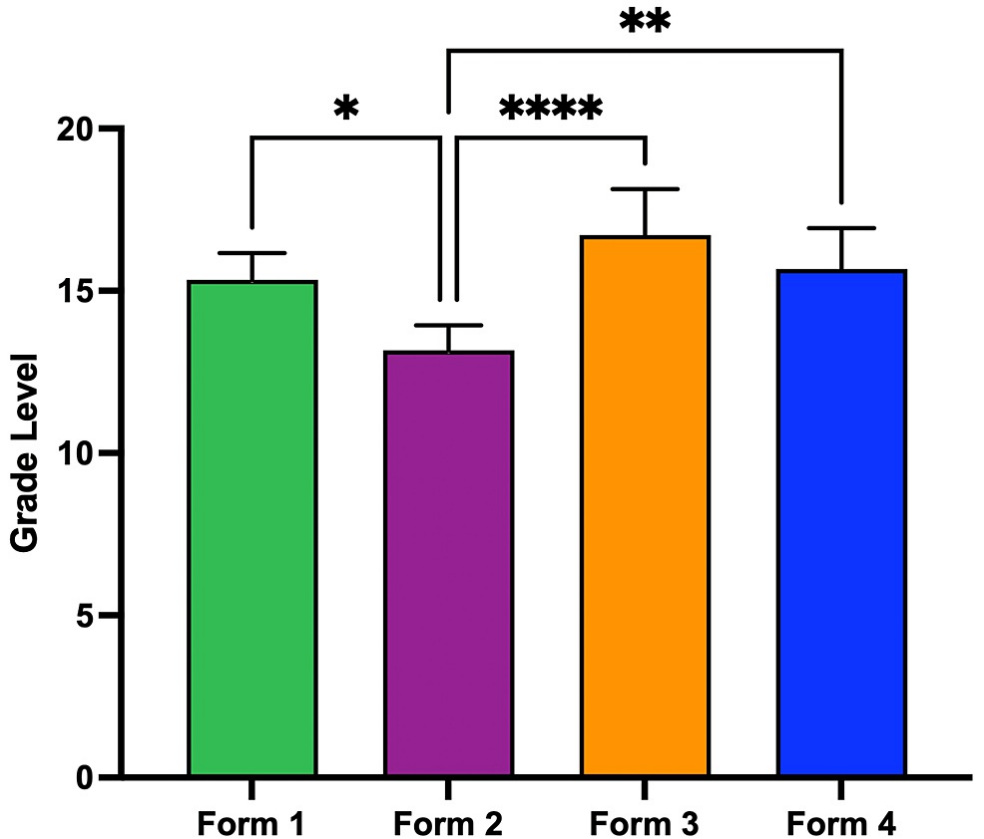
Mean grade level by form. Mean grade level of ChatGPT responses by form. * = p < 0.05; ** = p < 0.01; *** = p < 0.0001; **** = p < 0.0001.

**Figure 3:**
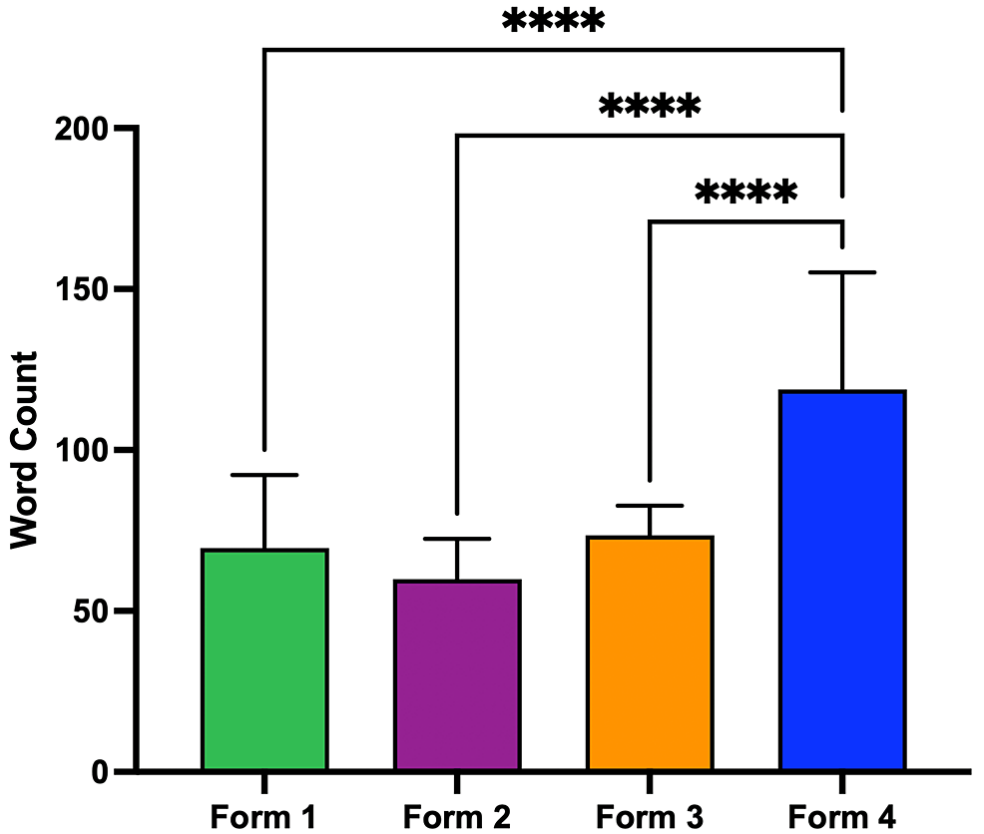
Mean word count by form. Mean word count of ChatGPT responses by form. * = p < 0.05; ** = p < 0.01; *** = p < 0.0001; **** = p < 0.0001.

The mean grade level was 14.23 ± 2.34 for Form 1, 12.81 ± 3.38 for Form 2, 16.73 ± 2.65 for Form 3, and 14.85 ± 2.76 for Form 4 (Figure 2). Form 2 responses, patient-friendly prompts, showed a lower mean grade level (12.81 ± 3.38) compared to Forms 1, 3, and 4 (p < 0.05). The mean word count was 69.50 ± 22.72 for Form 1, 59.94 ± 12.49 for Form 2, 73.50 ± 9.27 for Form 3, and 118.80 ± 36.41 for Form 4 (Figure 3). Form 4 responses, prompting for statistics/references, were significantly longer (p < 0.0001).

Across all forms, references were provided in three (4.7%) answers. Notably, when prompted for sources or references, ChatGPT only provided sources on three responses out of the 16 (18.8%). Three total sources were provided: two sources were from academic websites and one source was from published literature. This variation further shows the adaptability of ChatGPT in tailoring its responses to different prompting styles. While responses from all forms combined provided a total of three perfect scores (correct with references), a disproportionate number of these came from the Form 4 prompts, which specifically requested statistics and references.

## Discussion

Overall, ChatGPT provided accurate and comprehensive answers to most questions about AF regardless of prompting. ChatGPT answered at least “correct” for 90.6% of responses and answered at least “partially correct” for 98.4% of responses. Given the increase in the utilization of ChatGPT and all AI chatbots, the overall accuracy can be seen as a positive for patient education. Patient populations have a wide spectrum of health literacy. Although a specific person will likely not directly state their health literacy, e.g., “grade level” as defined in this study, it can be anticipated that in the context of a conversation, patients may show a higher or lower level of health literacy.

The National Institutes of Health (NIH) and the American Medical Association (AMA) recommend that patient education materials be written between the sixth and eighth-grade reading level [6,7]. Our study found that ChatGPT responses exceeded this grade level recommendation, averaging an FK score of 14.66, a collegiate reading level. Prior studies assessing ChatGPT’s grade level when responding to healthcare-related questions have found similar results [8-10]. Compared to other online sources, ChatGPT’s readability is on par. Non-academic websites on AF education average a grade level of 11.64, and academic sites average 13.05 [11]. Given that ChatGPT has shown, to some degree, an ability to alter its complexity based on its input, we believe that the grade level has little to no disadvantage to patients. The alignment of ChatGPT’s reading levels with those of established online medical resources can be viewed as both an advantage and a drawback. On the positive side, it demonstrates the tool’s capability to generate content that matches the complexity and detail found in professional medical literature, which can facilitate advanced learning and professional use. Conversely, it is essential to recognize that one of the main goals of AI in healthcare is to improve accessibility and understanding for a wider audience, including patients with varying levels of health literacy.

Our findings support that ChatGPT alters the complexity of its responses depending on the context of the conversation. When prompted as a patient, ChatGPT provides lower grade level responses compared to no prompting or prompting as a physician or researcher. While these results still do not meet the recommended sixth or eighth-grade reading level recommendation, it is still significant that ChatGPT can alter its response complexity. Previous studies have shown ChatGPT’s ability to comprehend medical information at differing complexities; however, the ability of an AI chatbot to alter its complexity based on input remains somewhat undefined [12].

Of note, in 1.6% of responses, ChatGPT presented patients with false information. A downside to AI chatbots is the tendency to confidently present incorrect information, known as “AI hallucinations” [13,14]. This underscores the need for users, especially patients, to critically evaluate the information provided and consult healthcare professionals for confirmation. Of the references that were provided by ChatGPT, all were correct. However, it has been reported that up to half of all cited sources are falsified, but still presented confidently [15]. Falsified citations have been well-characterized in the literature, with even the developers of ChatGPT acknowledging this shortcoming [16,17]. Additionally, when ChatGPT encounters questions involving unrelated variables, it tends to provide lengthy responses despite minimal supporting evidence. Consequently, the extent of ChatGPT’s inaccuracies largely depends on the nature of the questions posed. Therefore, ChatGPT’s performance is best for a limited range of questions that rely on answers derived from the large pre-existing literature used in the training of the language model [18].

This study shows that ChatGPT provides an accurate and adaptive source of medical information. However, studying ChatGPT has inherent limitations. Patients could input medical questions in an infinite number of ways which may generate answers not seen in this study. Additionally, AI language bots often alter their responses based on previous inputs, which may alter the level of accuracy and comprehension seen in this study. Further research should include a wider range of questions and compare responses from different AI models.

## Conclusions

This study shows that ChatGPT has significant potential as a supplemental source for patient education. The chatbot answered with a high degree of accuracy with rare occurrences of misinformation. The ability of ChatGPT to adapt its response complexity based on conversational context is a notable strength, enabling it to cater to individuals with varying levels of health literacy. Despite its responses often exceeding the recommended sixth to eighth-grade reading levels, the tool shows promise in aligning with the complexities found in the professional medical literature. Given this high reliability and reader adaptability, it is reasonable for healthcare providers to recommend ChatGPT as an informational resource while acknowledging the small but possible risk of encountering incorrect data.
